# Measuring electromagnetic fields (EMF) around wind turbines in Canada: is there a human health concern?

**DOI:** 10.1186/1476-069X-13-9

**Published:** 2014-02-15

**Authors:** Lindsay C McCallum, Melissa L Whitfield Aslund, Loren D Knopper, Glenn M Ferguson, Christopher A Ollson

**Affiliations:** 1 Department of Physical and Environmental Sciences, University of Toronto at Scarborough, Toronto, Ontario, Canada; 2 Intrinsik Environmental Sciences Inc, 500 - 6605 Hurontario Street, L5T 0A3, Mississauga, Ontario, Canada

**Keywords:** Electromagnetic fields, EMF, Wind turbines, Wind farms, Human health, Power lines, Transmission lines, Substation

## Abstract

**Background:**

The past five years has seen considerable expansion of wind power generation in Ontario, Canada. Most recently worries about exposure to electromagnetic fields (EMF) from wind turbines, and associated electrical transmission, has been raised at public meetings and legal proceedings. These fears have not been based on any actual measurements of EMF exposure surrounding existing projects but appear to follow from worries from internet sources and misunderstanding of the science.

**Methods:**

The study was carried out at the Kingsbridge 1 Wind Farm located near Goderich, Ontario, Canada. Magnetic field measurements were collected in the proximity of 15 Vestas 1.8 MW wind turbines, two substations, various buried and overhead collector and transmission lines, and nearby homes. Data were collected during three operational scenarios to characterize potential EMF exposure: ‘high wind’ (generating power), ‘low wind’ (drawing power from the grid, but not generating power) and ‘shut off’ (neither drawing, nor generating power).

**Results:**

Background levels of EMF (0.2 to 0.3 mG) were established by measuring magnetic fields around the wind turbines under the ‘shut off’ scenario. Magnetic field levels detected at the base of the turbines under both the ‘high wind’ and ‘low wind’ conditions were low (mean = 0.9 mG; n = 11) and rapidly diminished with distance, becoming indistinguishable from background within 2 m of the base. Magnetic fields measured 1 m above buried collector lines were also within background (≤ 0.3 mG). Beneath overhead 27.5 kV and 500 kV transmission lines, magnetic field levels of up to 16.5 and 46 mG, respectively, were recorded. These levels also diminished rapidly with distance. None of these sources appeared to influence magnetic field levels at nearby homes located as close as just over 500 m from turbines, where measurements immediately outside of the homes were ≤ 0.4 mG.

**Conclusions:**

The results suggest that there is nothing unique to wind farms with respect to EMF exposure; in fact, magnetic field levels in the vicinity of wind turbines were lower than those produced by many common household electrical devices and were well below any existing regulatory guidelines with respect to human health.

## Background

Wind power has been harnessed as a source of electricity around the world for decades and reliance on this form of energy is increasing. Despite its long standing history in other parts of the world, use of wind energy is relatively new in Canada [1]. While public attitude is generally overwhelmingly in favor of wind energy in the province of Ontario, with polls suggesting that support for wind energy is high (89% ‘supported’ or ‘somewhat supported’ wind energy in their region) [2], this support does not always translate into local acceptance of wind projects. Opposition to local wind projects has been particularly strong in Ontario, where wind turbines are becoming increasingly common in rural areas with over 1,500 MW installed since 2006 and another 2,800 MW expected to be installed by 2015 [3].

This local opposition has led to a number of legal appeals, via the Environmental Review Tribunal (ERT) process in Ontario, of the Renewable Energy Approvals (REA) granted to individual wind energy projects by the Ontario Ministry of the Environment (MOE). Since 2010, over 19 ERTs have either been completed or are in progress in Ontario [4]. Under the current legal framework for wind energy development in Ontario, REAs can be appealed by any member of the public on two grounds: 1) proceeding with the project will cause serious harm to human health and 2) proceeding with the project will cause serious and irreversible harm to plant life, animal life or the natural environment. At the time of publication of this article, no appeals have been successful on the basis of serious harm to human health and in a number of cases, electromagnetic fields (EMF) from the projects have been posited by appellants as the cause of serious harm to human health (e.g., GREP, Erickson, Ostrander) [5-7]. Although to date these appeals have been unsuccessful, concerns about the human health effects of wind turbines and EMF persist for some. The authors spend a considerable amount of time at public information sessions for projects and EMF is frequently raised as a health concern by the public.

The issue of EMF exposure and potential health effects predates the prevalence of wind energy in Canada. Early studies of residential exposure to EMF suggested a higher incidence of leukemia and brain cancer in children living near power lines having high wire configuration; however, more recent studies, which have improved upon the methods previously used, have been at best inconsistent [8]. The International Agency for Research on Cancer (IARC), an agency of the World Health Organization (WHO), has categorized EMF as a Class 2B possible human carcinogen, based on a weak association of childhood leukemia and chronic exposure to magnetic field strength above 3–4 mG [9]. This classification is based on the fact that there is limited evidence of carcinogenicity in humans and inadequate evidence of carcinogenicity in experimental animals. The human studies are weakened by various methodological problems that the WHO has identified as a combination of selection bias, some degree of confounding and chance [10]. There are also no globally accepted mechanisms that would suggest that low-level exposures are involved in cancer development. Thus, the WHO has stated (based on approximately 25,000 articles published over the past 30 years) that the evidence related to childhood leukemia is not strong enough to be considered causal [11].

There is a growing list of self-reported health symptoms that some individuals attribute to wind turbines specifically with respect to audible noise, low frequency noise and infrasound, shadow flicker and EMF. A study published in 2013 by Chapman et al., has reported over 200 symptoms, for example (but not limited to) difficulty sleeping, fatigue, depression, irritability, aggressiveness, cognitive dysfunction, nausea, dizziness, tinnitus, skin irritations, nosebleeds ringing in ears, headaches, lack of concentration, vertigo and sleep disruption [12]. In 2011, Havas and Colling claimed that exposure to EMF from wind turbines could be the cause this myriad of health issues in individuals considered to have ‘Electrohypersensitivity’ [13]; however, nowhere in their publication did Havas and Colling provide measured levels of EMF surrounding active wind turbines. Similar claims are frequently repeated on the internet. Although the relationship between these health issues and audible noise, low frequency noise and infrasound has been investigated in the scientific literature [14-24], limited research has been conducted with respect to EMF and wind turbines. Indeed, we are aware of only one study [25] where some characterization of EMF in proximity to wind turbines was reported. Israel et al. (2011) measured EMF levels 2 to 3 m from a wind energy park in Bulgaria consisting of 55 Vestas V90 3 MW towers and just outside nearby villages. The authors found that EMF was either below detection or was so small as to be considered “insignificant compared to the values found in other measurements in residential areas and homes” [25]. In their study, the EMF levels were measured between 0.133 and 0.225 mG. These values are well below the International Commission on Non-Ionizing Radiation Protection (ICNIRP) guideline of 2,000 mG for the protection of health of the general public.

This study was conducted to characterize EMF (as magnetic flux density) in the vicinity of an active wind farm in Ontario to address the heightened anxiety by some around EMF, wind turbines and human health. Measurements were taken at distances ranging from 0 to 500 m from turbines, and were collected under three operating conditions (i.e., turned on and generating power (high wind), turned on, drawing power and not generating power (low wind), and turned off and not drawing power from the grid (shut off)). Measurements were also collected in the vicinity of below and above ground electrical infrastructure (collector lines and substation), a 500 kV transmission line, and outside of a number of local homes in the wind farm area. Results are compared to EMF levels commonly encountered elsewhere in Canada and to existing guidelines.

## Methods

The study was carried out at the Kingsbridge 1 Wind Farm located near Goderich, Ontario, Canada. Spot measurements of magnetic field (i.e., magnetic flux density measured in units of milliGauss or mG) were obtained using a factory calibrated F.W. Bell ELF Gauss/Tesla Meter (model number 4180). The technical specifications of this meter include a minimum resolution of 0.1 mG and a measuring range of 0.1 mG to 599 mG with an accuracy of ± 2%. The field study, including equipment, standard measurement methodologies (e.g., 1 m above ground), and other considerations (e.g., distance, humidity, multiple sources), was developed in accordance with international protocols such as the Institute of Electrical and Electronics Engineers (IEEE) “Standard Procedures for Measurement of Power Frequency Electric and Magnetic Fields from AC Power Lines” [26-28]. All measurements were collected in 3-axis mode (XYZ), which provides a summation of the maximum magnetic flux density from all three dimensions surrounding the meter, and offers an indication of overall magnetic field level. For each measurement, the EMF meter was held 1 m above ground level and was allowed to stabilize for 5 seconds before the highest reading was recorded. Approximately 10% of measurements were collected in duplicate for quality assurance and control.

**Figure 1:**
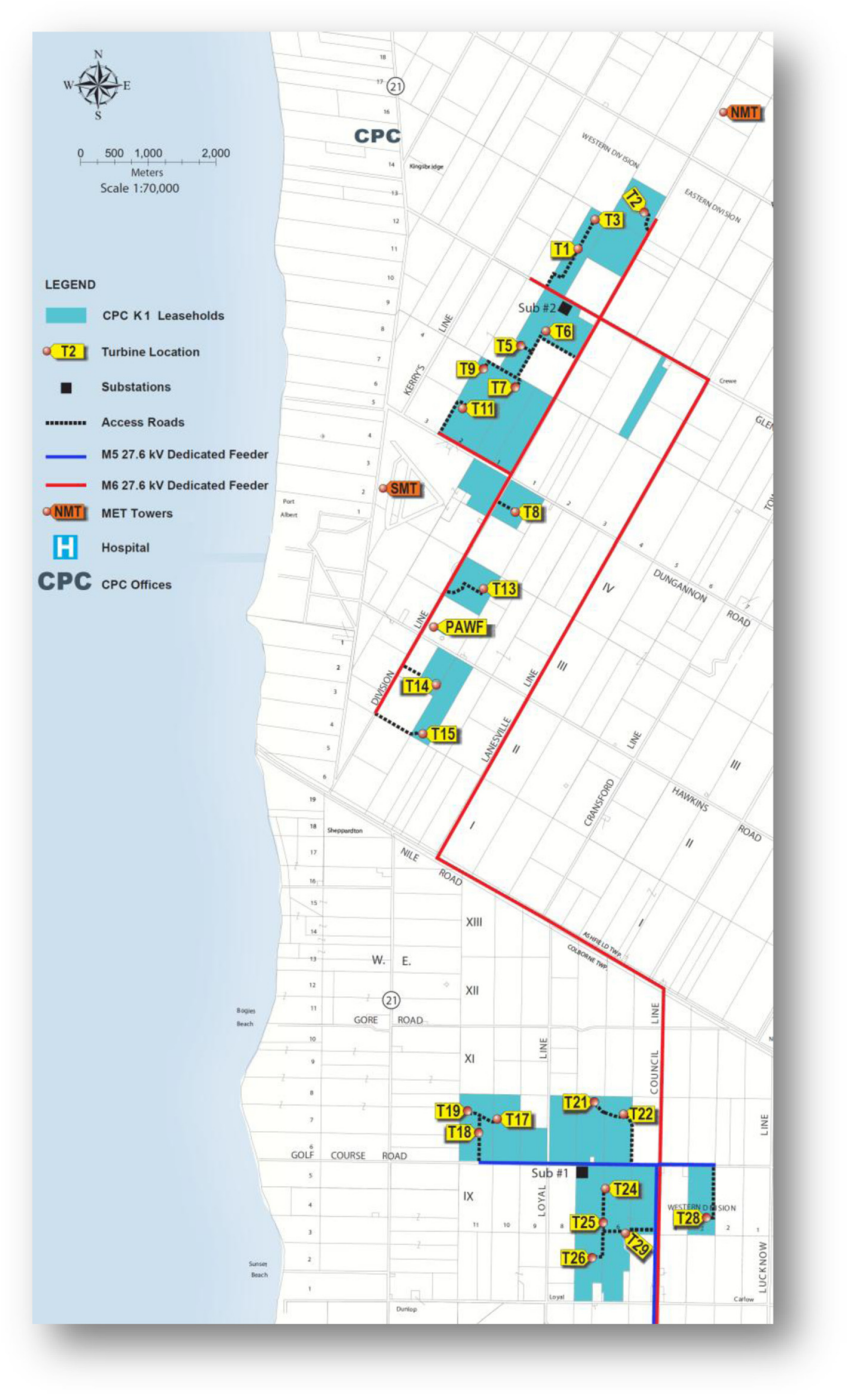
**Map of the study area.** The Kingsbridge (K1) Wind Farm near Goderich, Ontario, Canada. Included are the two substations, collector lines and turbine locations (samples collected around T1-T3, T6, T7, T11, T17, T18, T22, T25 and T29). Samples were also collected around T19, which was non-operational (not connected to the grid) and was used in this study as a control.

Magnetic field measurements were taken in the vicinity of 15 Vestas 1.8 MW wind turbines (Figure 1). One of the turbines was non-operational; this allowed measurements that could be used as a control. For each of the 15 turbines, the same series of measurements were taken. An initial reading was taken at the base of each turbine near the access door and another reading was taken 0.5 m away from the base, on the opposite side of the underground collector line. Subsequent measurements were taken at 2 m, 5 m, 10 m, 50 m, 100 m, 150 m and 200 m from the turbine. In a few instances, the surroundings allowed us to measure magnetic field levels at greater distance (i.e., up to 500 m) from the base of the turbine. Distances from the turbines were measured using a rangefinder (Cabela’s 800 by Bushnell). All of the turbines were located on agricultural land and surrounded by crops.

Measurements were collected under three different operational scenarios. In the first scenario (‘high wind’), measurements were collected when the wind was blowing at a sufficient speed to rotate the turbine blades and allow for power generation. In the second scenario (‘low wind’), the measurements were taken when the wind speed was insufficient to generate power, but the turbine was drawing power from the grid to ensure general maintenance and operations. For the third scenario (‘shut off’), measurements were collected when the turbines and associated collector lines were powered off completely.

In addition to the turbines, readings were taken above the buried collector lines (27.5 kV) for each turbine, beneath the overhead power lines (27.5 kV) and at the two wind farm substations. In addition, measurements were taken at the 500 kV line running from the Bruce Nuclear plant through the wind farm. For the 500 kV line, measurements were taken 1 m above ground moving away from the line at 5 m increments until background levels (0.2-0.3 mG) were reached. EMF readings were also taken immediately outside of seven project-participating homes (with landowner permission) in the study area that were 512–656 m to the closest wind turbine.

All magnetic field measurements were collected between 8 am and 6 pm on July 29^th^ and 30^th^, 2013. Measurements associated with the high wind scenario were collected on the first day since wind conditions in the area were ideal for power generation (average wind speed of 5.4 m/s; range = 3.3 – 7.6 m/s). The low wind and shut off scenario measurements were collected on the second day when wind speeds were lower (average speed 3.3 m/s; range = 0.2 – 4.9 m/s). The temperature for both days ranged from 15-21°C and weather conditions varied from overcast and rainy to sunny over the course of the study, with a relative humidity at 3.5 m above ground surface of 76% on July 29th and 69% on July 30th. All wind speed and temperature data for the study were provided by Zephyr North from two meteorological (MET) towers in the area [29].

## Results

Over 600 magnetic field measurements were collected at various distances from the wind turbines, homes, collector/transmission lines, and substations within the Kingsbridge 1 Wind Farm near Goderich, Ontario. Out of the 15 turbines measured, three were excluded since they were located in close proximity to other sources of EMF that caused interference (e.g., 500 kV transmission line), and one turbine was measured as a control since it is still standing but no longer operational. Where duplicate measurements were taken, the higher of the two values was used in the data analysis to maintain conservatism. There was excellent agreement between the duplicate samples, with readings either being identical or varying by ± 0.1 mG.

**Figure 2:**
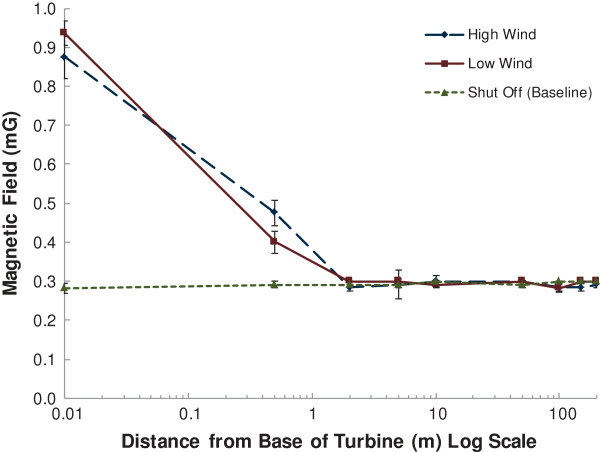
**Magnetic fields measured at various distances (log scale) from wind turbines under three different operational scenarios.** Mean magnetic field values have been provided (n = 11) in milligauss (mG). Error bars represent standard error of the mean. The three operational scenarios include turbines generating power (high wind), turbines not generating power but still connected to the power grid (low wind), and turbines and collector lines shut off completely (no wind).

Measurements taken around the turbines under the ‘shut off’ scenario were considered representative of baseline or background conditions given that they were not located in the proximity of any other known EMF sources. This baseline value was approximately 0.3 mG, regardless of distance from the turbines (Figure 2). Similar values (ranging from 0.2 to 0.3 mG) were also observed in proximity to the control (non-operational) turbine. Higher levels (mean: 0.9 mG; maximum: 1.1 mG) were detected at the base of the turbine under both the ‘high wind’ and ‘low wind’ conditions, but as expected based on the inverse power law, these levels rapidly diminished with distance from the turbine, becoming indistinguishable from background within approximately 2 m of the base of the turbines (Figure 2). In one case (not shown) magnetic fields were measured out to 500 m from the turbine where they remained within background levels. The lack of difference in magnetic field levels between the turbines operating under ‘high wind’ (generating power) and ‘low wind’ (not generating power) scenarios suggests that the measured magnetic fields are related to the power drawn by the turbine for maintenance and operations, rather than due to electricity generated by the turbine when it is spinning. Simply put, the low level measurements of EMF immediately adjacent to the access door of the turbines at their base were the same irrespective of the operating condition of the turbine.

For the seven houses assessed in this study, magnetic field measurements taken immediately outside (within 1 m) of the homes were consistently 0.4 mG, with the exception of one house that was vacant and had no power connections (0.2 mG). It is believed that this slight elevation above background is related to EMF generated within the home (i.e., wiring and use of electric devices). This is based on the fact that measurements collected outside of a home with no power connection were within background levels (0.2 mG). Despite this slight difference, all of the measurements taken outside of homes were <0.5 mG and considered to be very low.

Magnetic fields were also measured immediately above the buried 27.5 kV collector lines associated with each of the wind turbines included in the study. The readings were taken 1 m above ground and were consistently within measured study area background levels (0.2-0.3 mG). The overhead lines (27.5 kV) running along various roadways where the collector lines from the turbines went above ground and connected to the substations were also measured at 8 locations within the study area. Immediately beneath the power lines, magnetic field levels ranged from 0.3-16.5 mG (mean = 4.1 mG) and decreased to background within 10–25 m.

**Figure 3:**
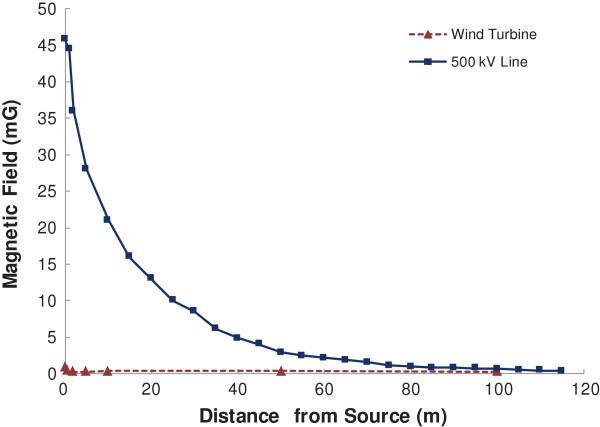
**Comparing magnetic fields around wind turbines and a 500 kV transmission line.** Mean magnetic field values have been provided (n = 11) in milligauss (mG). The ‘high wind’ turbine scenario is presented where conditions were sufficient for power generation.

Additionally, magnetic field measurements were collected immediately beneath the 500 kV transmission lines that run through the wind farm and are not at all associated with the wind project. Measurements were collected at various distances away until background levels were reached. Directly under the line, the magnetic field was approximately 46 mG, decreasing to 13 mG by 20 m, and reaching background (0.3 mG) by 115 m. The magnetic fields associated with the 500 kV power line were compared to the levels measured near wind turbines, where EMF levels immediately beneath the 500 kV line were almost 50 times higher than directly below the wind turbines operating under the ‘high wind’ scenario (Figure 3).

The two substations located within the study area were also measured to characterize potential magnetic field exposure. This was undertaken based on our awareness that there are a number of individuals that claim living nearby wind turbine project substations could adversely impact health. Each substation was surrounded by a metal fence; therefore, proximity measurements were limited to the fence line that was from 1.5 - 8 m away from the substation structure. This was considered acceptable since the fence prevents anyone from coming closer to the substation, thus fence line measurement would be the best way to characterize potential exposure. The magnetic field levels at the substations ranged from 0.2-4.1 mG when the turbines were operating under the ‘high wind’ scenario and ranged from 0.3-1.9 mG under the ‘shut off’ scenario.

## Discussion

EMF, radio waves, microwaves, visible light and x-rays are components of the electromagnetic spectrum. Each one of these forms of energy travels in waves and the strength of their energy is directly related to their wavelength [30]. For example, EMF associated with electricity is called extremely low frequency (ELF) because it is found below 300 Hz. In other words this type of energy moves at less than 300 waves per second. More specific to Canada, EMF associated with electricity is called power frequency EMF and travels at 60 Hz. ELF EMF has very little energy. In comparison, microwaves can travel at several billion waves per second and have enough energy to heat tissues.

Power frequency EMF are invisible lines of force that you cannot feel that surround electrical equipment, power cords, wires that carry electricity and outdoor power lines. Electric and magnetic fields can occur together or separately and are a function of voltage and current [30]. When an appliance is plugged into the wall, an electric field is present (there is voltage but no current); when that applicance is turned on, electric and magnetic fields are present (there is both voltage and current). Both electric and magnetic fields decrease with distance; however, electric fields are also dissipated by objects such as building materials, whereas magnetic fields can pass through most materials without being diminished. On a daily basis people around the world are exposed to ELF EMF as a result of using electricity [30].

To our knowledge this study is the first to provide quantitative measurements of EMF around wind turbines in Canada. One potential limitation of this study is that the transformers associated with the Kingsbridge 1 Wind Farm were located in the hub of the turbines, approximately 80 m above ground. There are a number of wind turbines that have pad mounted transformers located at ground level, which could potentially generate higher localized levels of EMF. However, preliminary data collected at a 110 Vestas V82 wind turbine with a pad mounted transformer from a nearby project location, suggests that although magnetic field levels tend to be higher at the base of the turbine transformer (67 mG), they drop off to background (0.2-0.3 mG) within 8 to 10 m. This indicates that despite the type of wind turbine (i.e., hub vs. pad mounted transformer) the EMF levels in the vicinity of wind turbines, especially at distances associated with typical residential setbacks, are considerably lower than the ICNIRP guideline for the general public (2,000 mG) [31].

**Figure 4:**
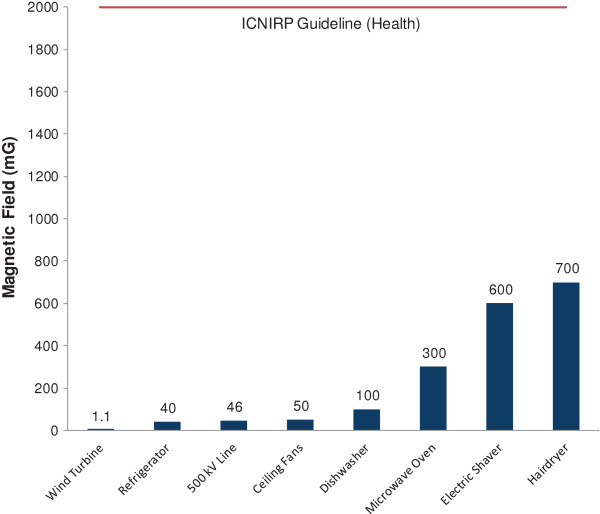
**Comparing magnetic fields from wind turbines and 500 kV power lines with common household electrical devices.** ‘Wind Turbine’ represents the maximum magnetic field (mG) measured at the base of wind turbines (n = 11) in the Kingsbridge 1 Wind Farm. ‘500 kV Line’ represents the maximum magnetic field measured beneath the 500 kV power line located within the study area. All other household electrical device data at six inch distance was taken from NIEHS [30]. The international regulatory standard published by ICNIRP [31] for EMF exposure protective of human health is provided for comparison.

Measurements collected in the vicinity of the 27.5 kV and 500 kV power lines were consistent with, if not lower than, those reported for typical 27.5 kV and 500 kV power lines by the US National Institute of Environmental Health Sciences (NIEHS). They report that a typical EMF level beneath a 500 kV line would be 86.7 mG, reducing to 1.4 mG at a distance of 91 m from the center of the line [30]. Additionally, the measurements taken at nearby homes (0.4 mG) are below the level that IARC originally used for the classification of EMF as a Class 2B possible human carcinogen (3–4 mG), which was based on limited evidence of carcinogenicity in humans and inadequate evidence of carcinogenicity in experimental animals [9]. Moreover, given the limited levels of EMF measured around the wind farm, human exposure to EMF from wind turbines is negligible in comparison to common household exposures. For example, typical magnetic field levels associated with common household appliances reported by the NIEHS at six inches from the source, include 40 mG for a refrigerator, 50 mG for a ceiling fan, 100 mG for a dishwasher, 300 mG for a microwave, 600 mG for an electric shaver and 700 mG for a hairdryer (Figure 4) [30].

Overall, our results support the official position of Health Canada, in that: “Health Canada does not consider that any precautionary measures are needed regarding daily exposures to EMFs at ELFs. There is no conclusive evidence of any harm caused by exposures at levels found in Canadian homes and schools, including those located just outside the boundaries of power line corridors” [32].

## Conclusions

The mean EMF level (characterized here by magnetic flux density) measured were 0.9 mG (n = 11) at the base of the wind turbines and dropped off to background levels (0.2-0.3 mG) within 2 m with levels consistently remaining at background out to 200 m and as far afield as 500 m. Additionally, magnetic fields measured at 1 m above buried collector lines were at background (0.2-0.3 mG), and readings taken below overhead 27.5 kV and 500 kV lines were consistent with other power distribution systems in North America. These results suggest that there is nothing unique to wind farms with respect to EMF exposure. In fact, magnetic field levels in the vicinity of wind turbines are lower than levels that people are exposed to on a daily basis in homes, offices and schools, and much lower than exposure we receive from many common household electrical devices (Figure 4). Our findings are consistent with those EMF measurements collected by Israel et al. (2011). Furthermore, when compared to ICNIRP guidelines, the levels of EMF measured around wind turbines were all well below levels known to cause harm to human health (Figure 4).

Collectively, these results suggest that the EMF surrounding wind turbines and their distribution systems (i.e., 27.5 and 500 kV power lines) are similar or lower than those commonly found throughout Ontario and across Canada. There was nothing unique about the EMF readings surrounding the wind turbines. Furthermore, the magnetic fields associated with power distribution systems, including those found in the vicinity of wind farms, are below levels that are expected to cause harm to human health based on international regulatory guidelines. Overall, our results do not support a potential causal link between power-frequency EMF and human health impacts at the low levels measured in the vicinity of the wind turbines.

## Abbreviations

ELF: Extremely low frequency; EMF: Electromagnetic Field; ERT: Environmental Review Tribunal; IARC: International Agency for Research on Cancer; ICNIRP: International Commission on Non‒Ionizing Radiation Protection; kV: kilovolt; MET: Meteorological; mG: milligauss; MOE: Ontario Ministry of the Environment; MW: Megawatt; NIEHS: US National Institute of Environmental Health Sciences; REA: Renewable Energy Approval; WHO: World Health Organization.

## Competing interests

In terms of competing interests (financial and non-financial), the authors work for a consulting firm and have worked with wind power companies. The study was funded in part by Capital Power, Samsung and Pattern and the authors are actively working in the field of wind turbines and human health. Dr. Ollson and Dr. Knopper have acted as expert witnesses for wind power companies during a number of legal hearings. Although we make this disclosure, we wish to reiterate that as independent scientific professionals our views and research are not influenced by these contractual obligations. The authors are environmental health scientists, trained and schooled, in the evaluation of potential health risks to people and the ecosystem through exposure to environmental issues such as wind turbines.

## Authors’ contributions

LCM, CAO and LDK designed the study methodology and LCM and CAO were responsible for data collection. LCM and MWA conducted the data analysis and interpretation. LCM researched and wrote the manuscript and all authors, including GMF in his oversight of the group, reviewed and approved the final version.
